# Deep immune profiling of MIS-C demonstrates marked but transient immune activation compared to adult and pediatric COVID-19

**DOI:** 10.1126/sciimmunol.abf7570

**Published:** 2021-03-02

**Authors:** Laura A. Vella, Josephine R. Giles, Amy E. Baxter, Derek A. Oldridge, Caroline Diorio, Leticia Kuri-Cervantes, Cécile Alanio, M. Betina Pampena, Jennifer E. Wu, Zeyu Chen, Yinghui Jane Huang, Elizabeth M. Anderson, Sigrid Gouma, Kevin O. McNerney, Julie Chase, Chakkapong Burudpakdee, Jessica H. Lee, Sokratis A. Apostolidis, Alexander C. Huang, Divij Mathew, Oliva Kuthuru, Eileen C. Goodwin, Madison E. Weirick, Marcus J. Bolton, Claudia P. Arevalo, Andre Ramos, CJ Jasen, Peyton E Conrey, Samir Sayed, Heather M. Giannini, Kurt D’Andrea, The UPenn COVID Processing Unit, Nuala J. Meyer, Edward M. Behrens, Hamid Bassiri, Scott E. Hensley, Sarah E. Henrickson, David T. Teachey, Michael R. Betts, E. John Wherry

**Affiliations:** 1 Division of Infectious Diseases, Department of Pediatrics, Children’s Hospital of Philadelphia, University of Pennsylvania Perelman School of Medicine, Philadelphia, PA, 19104, USA.; 2 Institute for Immunology, University of Pennsylvania Perelman School of Medicine, Philadelphia, PA, 19104, USA.; 3 Department of Systems Pharmacology and Translational Therapeutics, University of Pennsylvania Perelman School of Medicine, Philadelphia, PA, 19104, USA.; 4 Parker Institute for Cancer Immunotherapy at University of Pennsylvania Perelman School of Medicine, Philadelphia, PA, 19104, USA.; 5 Department of Pathology and Laboratory Medicine, Children's Hospital of Philadelphia, Philadelphia, PA 19104, USA.; 6 Department of Pathology and Laboratory Medicine, University of Pennsylvania Perelman School of Medicine, Philadelphia, PA 19104, USA.; 7 Immune Dysregulation Frontier Program, Department of Pediatrics, Children’s Hospital of Philadelphia, University of Pennsylvania Perelman School of Medicine, Philadelphia, PA, 19104, USA.; 8 Division of Oncology, Department of Pediatrics, Children’s Hospital of Philadelphia, University of Pennsylvania Perelman School of Medicine, Philadelphia, PA, 19104, USA.; 9 Department of Microbiology, University of Pennsylvania Perelman School of Medicine, Philadelphia, PA, 19104, USA.; 10 Division of Rheumatology, Department of Pediatrics, Children’s Hospital of Philadelphia, University of Pennsylvania Perelman School of Medicine, Philadelphia, PA, 19104, USA.; 11 Division of Rheumatology, Department of Medicine, University of Pennsylvania Perelman School of Medicine, Philadelphia, PA 19104, USA.; 12 Division of Hematology and Oncology, Department of Medicine, University of Pennsylvania Perelman School of Medicine, Philadelphia, PA 19104, USA.; 13 Division of Allergy and Immunology, Department of Pediatrics, Children’s Hospital of Philadelphia, University of Pennsylvania Perelman School of Medicine, Philadelphia, PA,19104, USA.; 14 Division of Pulmonary and Critical Care Medicine, Department of Medicine, University of Pennsylvania Perelman School of Medicine, Philadelphia, PA 19104, USA.; 15 Division of Translational Medicine and Human Genetics, Perelman School of Medicine at the University of Pennsylvania, Philadelphia, PA 19104, USA.

## Abstract

Pediatric COVID-19 following SARS-CoV-2 infection is associated with fewer hospitalizations and often milder disease than in adults. A subset of children, however, present with Multisystem Inflammatory Syndrome in Children (MIS-C) that can lead to vascular complications and shock, but rarely death. The immune features of MIS-C compared to pediatric COVID-19 or adult disease remain poorly understood. We analyzed peripheral blood immune responses in hospitalized SARS-CoV-2 infected pediatric patients (pediatric COVID-19) and patients with MIS-C. MIS-C patients had patterns of T cell-biased lymphopenia and T cell activation similar to severely ill adults, and all patients with MIS-C had SARS-CoV-2 spike-specific antibodies at admission. A distinct feature of MIS-C patients was robust activation of vascular patrolling CX3CR1+ CD8+ T cells that correlated with the use of vasoactive medication. Finally, whereas pediatric COVID-19 patients with acute respiratory distress syndrome (ARDS) had sustained immune activation, MIS-C patients displayed clinical improvement over time, concomitant with decreasing immune activation. Thus, non-MIS-C versus MIS-C SARS-CoV-2 associated illnesses are characterized by divergent immune signatures that are temporally distinct from one another and implicate CD8+ T cells in the clinical presentation and trajectory of MIS-C.

## Teaser

MIS-C is defined by lymphocyte activation, including elevated plasmablast frequencies and marked activation of CX3CR1+ CD8+ T cells.

## INTRODUCTION

Coronavirus disease 19 (COVID-19) from severe acute respiratory syndrome coronavirus 2 (SARS-CoV-2) infection is primarily an illness of adults, with morbidity and mortality increased with advanced age (*1*, *2*). In contrast, COVID-19 hospitalization is rare in children, accounting for <0.1% of total deaths (*3*–*5*). The reasons for differences in pediatric versus adult SARS-CoV-2 infection remain unclear given that other respiratory viruses can cause substantial morbidity and mortality in young children (*6*, *7*).

In adults, COVID-19 morbidity is associated with increased clinical markers of inflammation (*2*, *8*). Clinical measures of cellular immunity are limited, but decreased absolute lymphocyte counts (ALC) are a consistent finding and are associated with worse outcomes (*9*–*11*). Translational studies in adult COVID-19 identified T cell lymphopenia in particular, with a preferential decrease in CD8+ T cells compared to CD4+ T cells (*11*, *12*). In those CD4+ and CD8+ T cells remaining, marked activation was observed and correlated positively with the severity of illness (*11*–*14*). However, even among the sickest adults, the degree of immune activation varies (*12*–*15*). Reports of children hospitalized with COVID-19 suggest similar clinical inflammatory profiles, including elevated c-reactive protein (CRP), ferritin, procalcitonin (PCT), and reduced ALC (*16*, *17*). However, how these clinical inflammatory markers relate to cellular immune perturbations is unknown. It therefore remains unclear whether the outcome differences in children compared to adults are associated with distinct profiles of cellular immune activation, involvement of different immune cell types, or if differences are non-immunological and low pediatric mortality occurs despite a similar immune landscape.

After the initial wave of COVID-19 hospitalizations, children began to develop Multisystem Inflammatory Syndrome in Children (MIS-C), a syndrome commonly presenting with vascular involvement and shock (*18*–*24*). MIS-C is suggested to be a post-infectious or delayed-infectious event (*21*, *24*, *25*) and has similarities in clinical presentation to Kawasaki disease, especially the vascular involvement. However, MIS-C and Kawasaki disease differ in key clinical, inflammatory, and autoantibody signatures (*21*, *26*, *27*). MIS-C is associated with similar or higher clinical inflammatory markers than observed in adult and pediatric COVID-19 (*18*–*20*). Clinically, MIS-C has a distinct presentation compared to the respiratory manifestations more typical of SARS-CoV-2 infection. The immunologic features driving MIS-C remain poorly defined, but MIS-C may be associated with altered innate and adaptive cell frequencies, including a subtle loss of T cells and increased memory T cell activation compared to healthy pediatric controls (*26*, *28*, *29*).

We therefore interrogated the immune state in pediatric COVID-19 and MIS-C using deep immune profiling. We collected blood from patients admitted to the Children’s Hospital of Philadelphia in April through June of 2020 and performed high dimensional flow cytometry in parallel with samples from adult COVID-19 patients, recovered adult COVID-19 subjects, and healthy adults, as described (*12*, *14*). In children, these cellular analyses were paired with serologic and plasma cytokine data and integrated with clinical and laboratory information. Our results demonstrated that although the immune landscape in pediatric COVID-19 was similar to adults, MIS-C represented an exacerbated T cell activation state, particularly for CD8+ T cells, including a highly activated vascular patrolling CD8+ T cell subset. These features of profound T cell activation decreased concurrently with clinical improvement. Together, our findings provide a broad immunologic foundation for understanding pathogenesis and recovery in this novel SARS-CoV-2 associated inflammatory syndrome with potential implications for adult disease.

## RESULTS

### Cytopenias in MIS-C included enhanced T cell lymphopenia

**Fig. 1:**
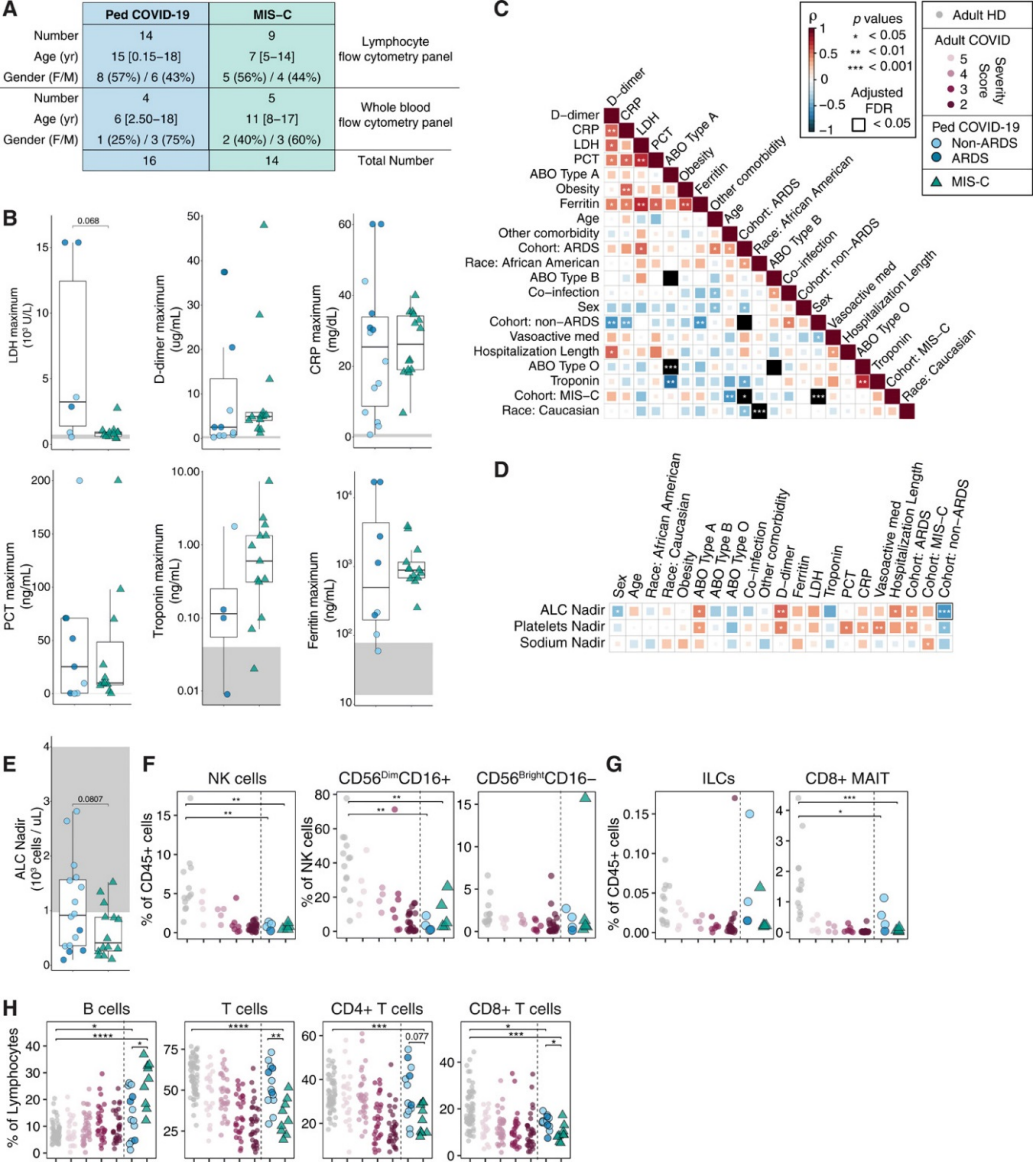
Cytopenias in MIS-C include enhanced T cell lymphopenia. (**A**) Overview of study cohorts. (**B**) Maximum clinical inflammatory markers. (**C**) Spearman correlation and hierarchical clustering of indicated clinical features and maximum laboratory values. Solid black boxes indicate mutually exclusive comparisons. (**D**) Spearman correlation of clinical parameters for which the lowest recorded (nadir) values are relevant for indicated features. (**E**) Nadir absolute lymphocyte counts (ALC). (**F**) Frequencies of total NK cells and NK subsets across adult healthy donors (HD), adult COVID-19, and pediatric cohorts. (**G**) Frequencies of ILCs (left) and CD8+ MAIT cells (right) across all cohorts. (**H**) Quantification of major lymphocyte populations across all cohorts. (**BEFGH**) Each dot represents an individual patient or subject, with adult HD in gray circles, adult COVID-19 in shades of mauve indicated by disease severity score, Ped COVID-19 in blue circles, with ARDS patients in dark blue, and MIS-C in green triangles. See key, top right. (**BE**) Normal laboratory reference ranges for healthy pediatric subjects are indicated in gray shading. Significance determined by unpaired Wilcoxon test between two pediatric groups for all clinical measures. P value shown when p <0.1, but >0.05. Lack of notation indicates no statistical significance. (**CD**) Spearman's Rank Correlation coefficient (ρ) is indicated by square size and heat scale; significance indicated by: * p<0.05, ** p<0.01, and *** p<0.001; black box indicates FDR<0.05 following Benjamini-Hochberg correction. See key, top right. (**FGH**) Significance determined by unpaired Wilcoxon test between adult HD and each pediatric group, and between pediatric groups, with adjustment for multiple comparisons using Benjamini-Hochberg correction, indicated by: * p<0.05, ** p<0.01, and *** p<0.001. Lack of notation for specified comparisons indicates no statistical significance. See Table S5 for subject numbers per panel.

We collected peripheral blood from patients admitted to the Children’s Hospital of Philadelphia from April through July 2020, and thirty patients had samples available for immunophenotyping. Of these 30, 16 were diagnosed with COVID-19 and 14 with MIS-C (Fig. 1A, Table S1). Of the patients with COVID-19, 4 were determined to have acute respiratory distress syndrome (ARDS, see Table S1 for patient characteristics). Patients with MIS-C were treated with intravenous immunoglobulin (IVIg) and most received steroids. Patients with COVID-19 received immune modulation when clinically indicated (Table S1). The timing of immune modulation relative to research blood draw(s) is indicated in Figure S1.

Consistent with previous reports (*18*–*20*, *24*), the maximum values for clinical measures of inflammation varied but were elevated in most subjects (Fig. 1B). Although inflammatory markers in COVID-19 and MIS-C were comparable, measures of inflammation were more often obtained for patients admitted to the intensive care unit (ICU) and for patients with MIS-C; as a result, patients with less severe COVID-19 are underrepresented (Table S1). We next investigated the relationships between clinical markers of inflammation, demographics, and disease severity measures including the need for vasoactive medications (Fig. 1C). The diagnosis of MIS-C, although less likely in someone with comorbidities, was not distinguished from pediatric COVID-19 (ARDS or non-ARDS) by correlation with laboratory measures, with the exception of hyponatremia, which was nominally correlated with MIS-C (Fig. 1D). Further, the white blood cell counts and associated differentials were variable (Fig. S2A, 2B), as was the lymphocyte nadir (Fig. 1E). Nevertheless, both the ALC and platelet nadir had nominal correlations with clinical disease metrics, consistent with severe illness (Fig. 1D). Taken together, these clinical values suggested heterogeneity in pediatric patients with COVID-19 and MIS-C, with inflammatory marker elevation and cytopenias occurring in both groups.

We next performed high dimensional flow cytometry on whole blood or freshly isolated peripheral blood mononuclear cells (PBMC) (Fig. 1A, Tables S2 and S3) in parallel with adult samples from recently reported datasets (*12*, *14*) that were re-analyzed for integration with pediatric data. Among non-lymphocytes, as a proportion of CD45+ cells, eosinophils and immature granulocytes were similar between pediatric COVID-19 and MIS-C (Fig. S2C, S2D). Neutrophils tended to be increased in MIS-C by flow cytometric and clinical measurements, though this change was not statistically significant (Fig. S2B, S2C, S2D). Monocytes and DC were also comparable (Fig. S2B, S2C, S2E, S2F). However, plasmacytoid DC were decreased in MIS-C (Fig. S2C, S2F), consistent with previous studies in MIS-C (*28*) and adults (*13*, *14*).

Transient lymphopenia is a feature of viral infection (*30*, *31*) but is typically only observed at symptom onset and recovers quickly (*32*). In our cohort, lymphopenia was observed in most patients (Fig. 1E), consistent with previous studies (*17*–*20*, *24*, *29*, *33*, *34*). In adult COVID-19, lymphopenia is T cell biased (*11*, *12*, *35*). However, the relative impact on lymphocyte subsets and phenotype in pediatric COVID-19 and MIS-C has not been defined. We therefore interrogated B cells, CD4+ T cells, CD8+ T cells, mucosal associated invariant T cells (MAITs), and natural killer (NK) cells using high dimensional flow cytometry. Total NK cell and cytolytic NK subset (CD56^dim^CD16+) frequencies were reduced in both pediatric cohorts compared to healthy adults (Fig. 1F, S2C). Although MAITs were decreased in both pediatric cohorts compared to healthy adults, innate lymphocyte (ILC) frequencies appeared similar (Fig. 1G, S2C) (*14*). These findings are consistent with adult COVID-19 subjects. This interpretation may be limited by the absence of contemporaneously analyzed healthy pediatric samples which were not available during the early phase of the pandemic. Unlike adult COVID-19, pediatric COVID-19 was not consistently associated with reduced T cell frequencies (Fig. S2G, Fig. 1H). In contrast, patients with MIS-C displayed reduced T cell frequencies compared to healthy adults, particularly for CD8+ T cells. These data suggest that pediatric COVID-19 and MIS-C are both inflammatory disease presentations with concomitant lymphopenia, though the T cell bias of the lymphopenia in MIS-C is similar to severely ill adults.

### Increased T cell activation in MIS-C compared to pediatric COVID-19

**Fig. 2:**
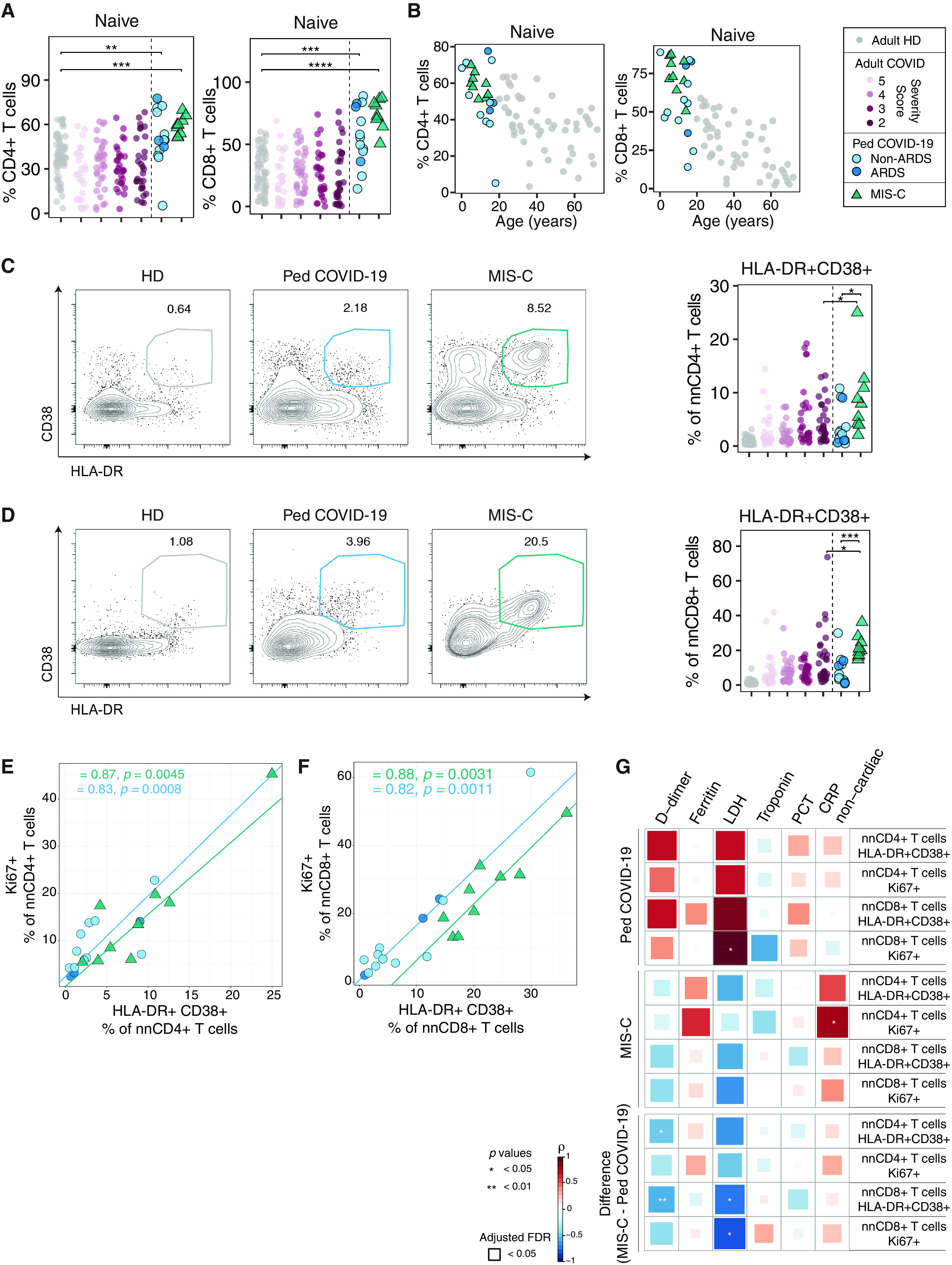
T cell activation in MIS-C was greater than in COVID-19. (**A**) Proportion of naive cells among CD4+ (left) and CD8+ (right) T cells in all cohorts. (**B**) Association between age and proportion of naive T cells for pediatric cohorts and adult HD. (**C**) Representative plots and quantification of HLA-DR+CD38+ non-naive (nn)CD4+ T cells. (**D**) Representative plots and quantification of HLA-DR+CD38+ non-naive (nn)CD8+ T cells. (**EF**) Correlation between Ki67+ and HLA-DR+CD38+ populations for nnCD4+ (**E**) and nnCD8+ (**F**) T cells in pediatric cohorts. (**G**) Spearman correlation for indicated clinical features with frequencies of HLA-DR+CD38+ and Ki67+ nnCD4+ and nnCD8+ T cells. Top and middle: correlations within each cohort (Ped COVID-19 and MIS-C). Bottom: difference in Spearman correlation between the two pediatric cohorts. (**A-F**) Each dot represents an individual patient or subject. See key, top right. (**A**) Significance determined by unpaired Wilcoxon test between adult HD and each pediatric cohort, and between pediatric groups, with adjustment for multiple comparisons using Benjamini-Hochberg correction, indicated by: * p<0.05, ** p<0.01, *** p<0.001. Lack of notation for specified comparisons indicates no statistical significance. (**CD**) Significance determined by unpaired Wilcoxon test between adult COVID Severity Score 2 and each pediatric group, and between pediatric groups, with adjustment for multiple comparisons using Benjamini-Hochberg correction, indicated by: * p<0.05, *** p<0.001. Lack of notation for specified comparisons indicates no statistical significance. (**EF**) Non-parametric trend lines (Theil-Sen) for each pediatric cohort shown, with Spearman’s Rank Correlation coefficient (ρ) and P value. (**G**) Spearman's Rank Correlation coefficient (ρ) indicated by square size and heat scale; (bottom) the difference in ρ between Ped COVID-19 and MIS-C, divided by 2 to normalize to a -1 to 1 scale; permutation test significance indicated by: * p<0.05, ** p<0.01. Absence of black box indicates failure to meet FDR<0.05 following Benjamini-Hochberg correction. See Table S5 for subject numbers per panel.

As expected (*36*), the frequency of naive CD4+ and CD8+ T cells was higher in the pediatric cohorts (Fig. 2A) and decreased with age (Fig. 2B). As a result, subsequent T cell analyses are based on frequencies of non-naive (nn) subsets. For both CD4+ and CD8+ T cells, the frequency of central memory (CM, CD45RA-CD27+CCR7+) and the effector memory 1 (EM1, CD45RA-CD27+CCR7-) subsets was similar between adult and pediatric cohorts, whereas the CD8+ effector memory 2 (EM2, CD45RA-CD27-) subset was slightly lower in children (Fig. S3A-C). As expected (*37*), terminally differentiated effector memory T cells RA (EMRA, CD45RA+ CD27- CCR7-) were present at a lower frequency in our pediatric cohorts (Fig. S3B, S3C). The naive, effector, and central memory subset distributions were not different between the pediatric COVID-19 and MIS-C (Fig. S3A-C).

T cell activation and proliferation are hallmarks of acute viral infections (*38*) (*11*, *12*, *14*). Pediatric COVID-19 patients had marked proliferation in CD4+ T cells, with a similar range of Ki67+ CD4+ T cells as adult COVID-19 patients (Fig. S3D). CD4+ T cell proliferation in MIS-C patients was similar to pedCOVID, but elevated compared to severe adult disease (Fig. S3D). In contrast, there were more Ki67+ CD8+ T cells in MIS-C compared to pediatric COVID-19 (Fig. S3E). Moreover, the proliferation of CD8+ T cells in MIS-C exceeded that observed for the majority of adults with COVID-19 (Fig. S3E). The co-expression of HLA-DR and CD38 can identify recently activated T cells responding to viral infection (*12*, *39*–*41*). MIS-C had higher frequencies of HLA-DR+CD38+ CD4+ and CD8+ T cells compared to pediatric COVID-19, again rivaling or exceeding that observed in adult COVID-19 (Fig. 2C-D). Among the pediatric COVID-19 patients, those with ARDS were not obviously different in T cell activation compared to non-ARDS patients, though the number of ARDS patients was low.

We next examined T cell activation and proliferation in T cell subsets. In general, patterns were consistent for pediatric COVID-19 and MIS-C and mirrored those in adults. Proliferation and activation were most robust for EM1 CD8+ T cells and CM CD4+ T cells (Fig. S3F, S3G). Despite heterogeneity in the range of Ki67+ or HLA-DR+CD38+ cells, proliferation and activation were positively correlated in both pediatric COVID-19 and MIS-C (Fig. 2E, 2F). These data indicated that pediatric SARS-CoV-2 infection results in robust T cell proliferation and activation. Moreover, children with MIS-C displayed marked T cell activation and proliferation, especially for CD8+ T cells, even compared to severely ill adults.

In addition to conventional lymphocytes, innate and innate-like lymphocytes become activated during human viral infection and vaccination (*42*–*44*). In MIS-C, more than 80% of NK cells were CD38+ (Fig. S4A). A higher frequency of CD38+ cells in MIS-C compared to pediatric COVID-19 was also observed for MAIT cells (Fig. S4B) (*42*, *45*). These data suggest that MIS-C is associated with activation of innate lymphocytes in addition to conventional CD4+ and CD8+ T cells.

We next investigated the relationships between lymphocyte activation and proliferation with clinical measures of inflammation and disease. In pediatric COVID-19, there was a positive relationship between many clinical markers of inflammation and T cell activation, though only the correlation between Ki67+ CD8+ T cells and LDH achieved nominal significance (Fig. 2G, top panel). In contrast, in MIS-C, the relationships between T cell activation and inflammation trended in the opposite direction (Fig. 2G, middle), although these associations were not significant. Given the difference between groups, we next asked how these relationships between T cell activation and clinical features compared between MIS-C and pediatric COVID-19. This analysis revealed a difference in how T cell activation was correlated with D-dimer or LDH in the two diseases (Fig. 2G, bottom) and further supported the notion that immune activation in MIS-C manifests with different relationships with clinical inflammation compared to pediatric COVID-19.

### Distinct activation of CD8+ T cell populations associated with persistent antigen and vascular surveillance in MIS-C

**Fig. 3:**
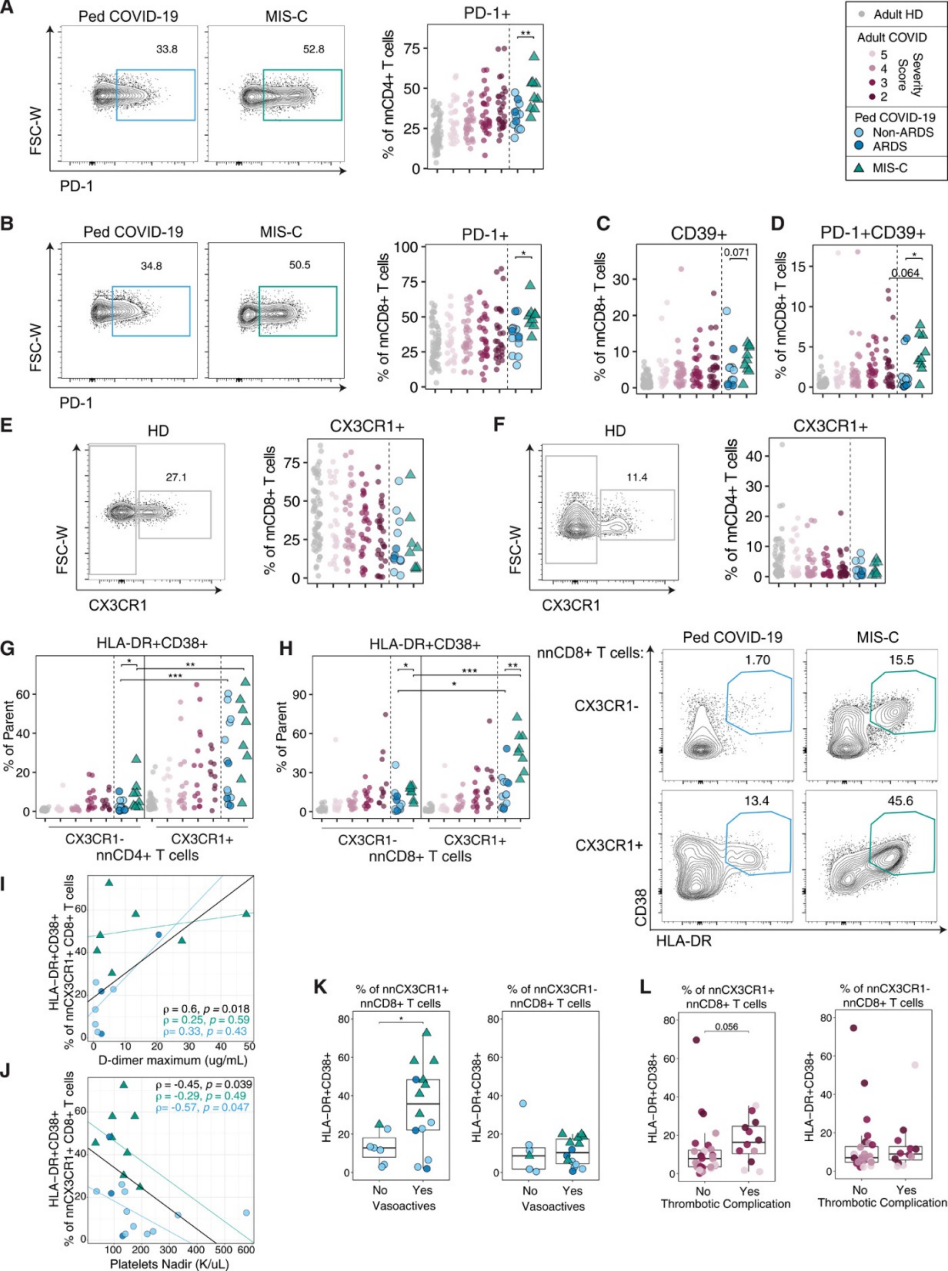
MIS-C was uniquely marked by activation in CD8+ T cell populations associated with persistent antigen and vascular surveillance. (**AB**) Representative plots and quantification of PD-1+ nnCD4+ T cells (**A**) and nnCD8+ T cells (**B**). (**C**) Frequencies of CD39+ nnCD8+ T cells. (**D**) Frequencies of CD39+PD-1+ nnCD8+ T cells. (**EF**) Representative plots and quantification of CX3CR1+ nnCD8+ (**E**) and nnCD4+ T cells (**F**). (**G**) HLA-DR+CD38+ frequencies within CX3CR1- and CX3CR1+ nnCD4+ T cells. (**H**) Representative plots and quantification of HLADR+CD38+ population within CX3CR1- and CX3CR1+ nnCD8+ T cells. (**IJ**) Frequency of HLA-DR+CD38+ CX3CR1+ nnCD8+ T cells versus maximum D-Dimer (**I**) and platelet nadir (**J**). (**K**) Frequencies of HLA-DR+CD38+ CX3CR1+ (left) and CX3CR1- (right) nnCD8+ T cells in pediatric patients categorized by treatment with vasoactive medications. (**L**) Frequencies of HLA-DR+CD38+ CX3CR1+ (left) and CX3CR1- (right) nnCD8+ T cells in adult COVID-19 patients categorized by thrombotic complication. (**A-L**) Each dot represents an individual patient or subject. See key, top right. (**A-C, E-F**) Significance determined by unpaired Wilcoxon test between Ped COVID-19 and MIS-C groups only, indicated by: * p<0.05, ** p<0.01. (**D**) Significance determined between each pediatric group and adult COVID Severity Score 2 with adjustment for multiple comparisons using Benjamini-Hochberg correction, indicated by: * p<0.05 or P value shown where p >0.05 but <0.1. (**GH**) Significance determined by unpaired Wilcoxon test between Ped COVID-19 and MIS-C groups; or by paired Wilcoxon test between CX3CR1- and CX3CR1+ within pediatric groups, with adjustment for multiple comparisons using Benjamini-Hochberg correction, indicated by: * p<0.05, ** p<0.01, *** p<0.001. (**IJ**) Non-parametric trend lines (Theil-Sen) for total pediatric cohort (black), MIS-C (green) and pedCOVID-19 (blue), with Spearman’s Rank Correlation coefficient (ρ) and P value. (**KL**) Significance determined by unpaired Wilcoxon test between clinical category, indicated by: * p<0.05 or P value shown where p >0.05 but <0.1. Lack of notation for specified comparisons indicates no statistical significance. See Table S5 for subject numbers per panel.

In MIS-C, one possible mechanism of immune perturbation is chronic antigen exposure driving immune dysfunction or exhaustion (*46*). Because MIS-C is thought to present clinically ~3-4 weeks after exposure to SARS-CoV-2, viral RNA positivity in most MIS-C patients in this cohort (Table S1) suggests continued antigen availability. To explore this possibility, we examined markers associated with exhaustion. The inhibitory receptor PD-1 is expressed by exhausted T cells but can also be expressed by recently activated T cells (*47*–*50*), and in CD4+ T cells PD-1 is also a marker of T follicular helper cells (Tfh) (*51*). In adult COVID-19, PD-1 expression by CD4+ T cells was elevated (*12*). For most pediatric patients, the frequency of PD-1+ CD4+ T cells was similar to adults; however, PD-1+ CD4+ and CD8+ T cell frequencies were substantially higher in MIS-C compared to pediatric COVID-19 (Fig. 3A, 3B). To better assess CD8+ T cell exhaustion, we used CD39, where co-expression of PD-1 and CD39 is strongly associated with exhausted or chronically stimulated CD8+ T cells (*52*–*54*). Although CD39 expression by CD8+ T cells alone was not significantly elevated (Fig. 3C), the co-expression of both PD-1 and CD39 by CD8+ T cells was substantially increased in MIS-C compared to pediatric COVID-19 (Fig. 3D). The frequency of PD-1+CD39+ CD8+ T cells in MIS-C was high even compared to severely ill adults, though this comparison was not statistically significant after corrections for multiple comparisons. Although further studies are needed, increased frequency of PD-1+CD39+ CD8+ T cells in MIS-C supports a role for prolonged antigen stimulation in the inflammatory syndrome.

Given the vascular aspects of MIS-C, we next investigated whether MIS-C was associated with changes in CD8+ T cells that express the fractalkine receptor CX3CR1. CX3CR1+ CD8+ T cells can interact with and adhere to fractalkine-expressing activated endothelium, and these interactions foster the ability of CX3CR1+ CD8+ T cells to patrol vasculature (*55*–*58*). As previously described, CX3CR1+ CD8+ T cells were most abundant in EMRA subsets in adults as compared to EM1 subsets in children, with all cohorts demonstrating increased T-bet in CX3CR1+ CD8+ T cells (Fig S5A, Fig S5B) (*58*–*60*). The overall frequencies of CX3CR1+ CD4+ and CD8+ T cells were not increased in MIS-C compared to pediatric COVID-19 (Fig. 3E, Fig. 3F). However, CX3CR1+ CD4+ and CD8+ T cells were both more highly activated and proliferating than CX3CR1- CD4+ and CD8+ T cells, based on CD38 and HLA-DR (Fig. 3G, Fig. 3H) or Ki67 expression (Fig. S5C, Fig. S5D). Moreover, compared to pediatric COVID-19, CX3CR1+ CD8+ T cells in MIS-C were markedly more activated, and a substantially higher proportion were Ki67+ and PD-1+ (Fig. 3H, Fig. S5C, Fig. S5E). Although CX3CR1- T cells were also more activated in MIS-C, the degree of activation and proliferation in CX3CR1+ CD8+ T cells in MIS-C suggested a potential role for increased CD8+ T cell vascular interactions in MIS-C compared to pediatric COVID-19 patients.

Although activation of CX3CR1+ CD8+ T cells was highest in MIS-C, this pattern was also observed in some non-MIS-C pediatric and adult COVID-19 patients. Given these observations and the associations between fractalkine and vascular inflammation (*55*, *61*–*63*), we next assessed whether activated CX3CR1+ T cells correlated with vascular presentations of disease. When all pediatric patients were assessed together for the coagulation-associated measures of D-dimer and platelets, activation of CX3CR1+ CD8+ T cells was positively correlated with D-dimer and inversely correlated with platelet nadir (Fig. 3I, Fig. 3J). To further investigate a relationship between activated CX3CR1+ CD8+ T cells and vascular disease, we next asked whether the frequency of activated CX3CR1+ CD8+ T cells was associated with need for vasoactive support in pediatric SARS-CoV-2 infection. Indeed, patients requiring vasoactive medication had substantially more activated CX3CR1+ CD8+ T cells, but no such relationship existed for activated CX3CR1- CD8+ T cells (Fig. 3K). This relationship was driven by MIS-C status, but the association was observed in all groups. We then asked whether these observations in children might inform our understanding of adult COVID-19 disease and coagulopathy. Most adults had activated CX3CR1+ CD8+ T cell frequencies below that observed in MIS-C. Nevertheless, the frequency of activated CX3CR1+ CD8+ T cells was higher (*P*=0.056) in adult COVID-19 patients with suspected or confirmed thrombotic complications (Fig. 3L). This difference was not observed for CX3CR1- CD8+ T cells (Fig. 3L). These data suggested that MIS-C is associated with distinct features of T cell activation and identify a potential relationship between the activation status of vascular patrolling CD8+ T cells in the presentation of MIS-C and vascular complications of COVID-19 more broadly.

### Prolonged plasmablast responses in MIS-C

**Fig. 4:**
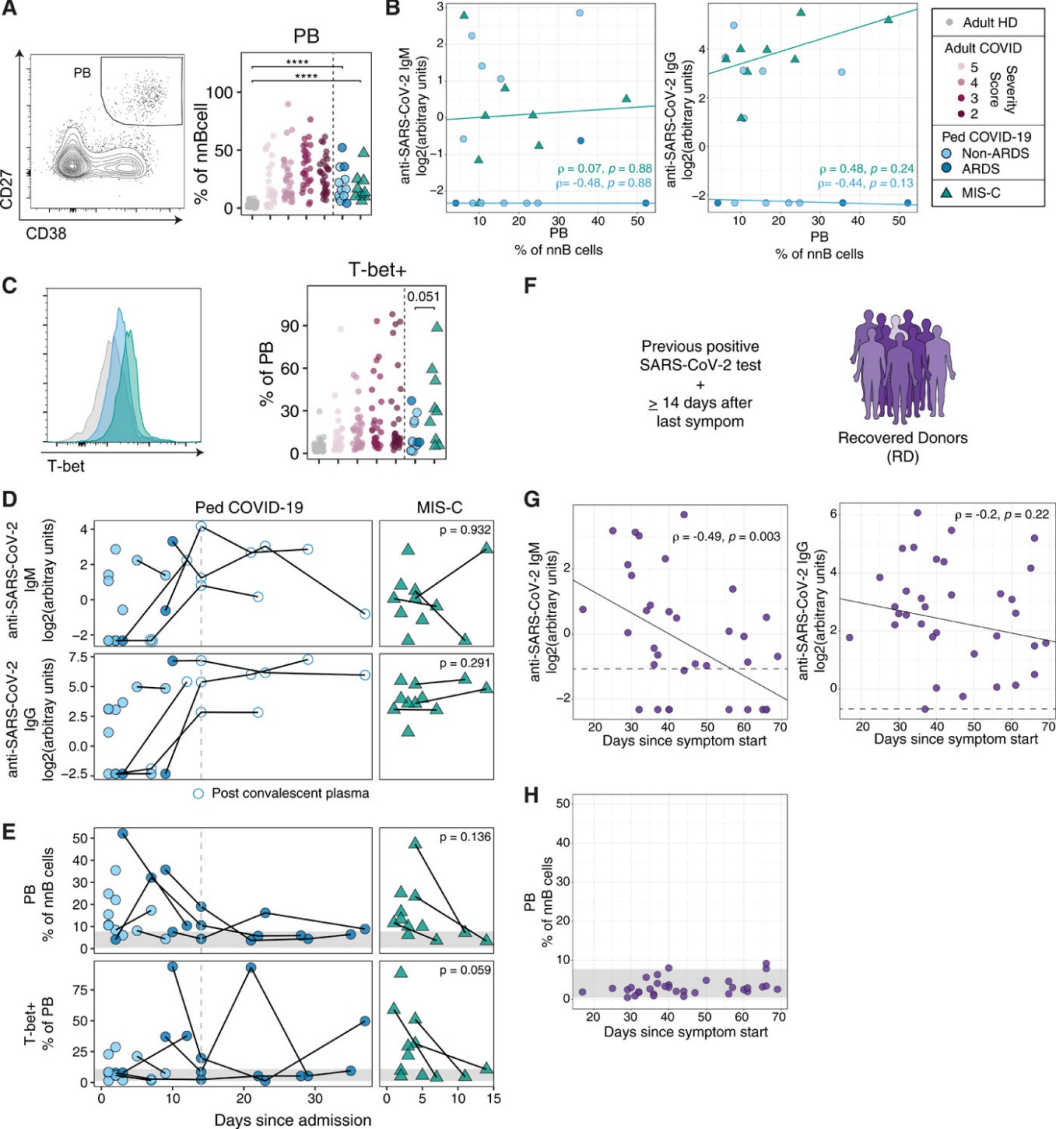
Prolonged plasmablast responses in MIS-C. (**A**) Representative plots and quantification of plasmablasts (PB). (**B**) Frequency of PB within non-naive (nn) B cells versus anti-SARS-CoV-2 IgM (left) and IgG (right). (**C**) Representative histogram of T-bet expression by PB for an adult HD (gray), Ped COVID-19 (blue) and MIS-C (green) and quantification of T-bet+ PB frequencies. (**D**) SARS-Cov-2 antibody levels and (**E**) B cell features over days since admission in Ped COVID-19 (left) and MIS-C subjects (right). Black lines connect repeat draws for individual subjects. (**F**) Overview of adult recovered donor (RD) cohort. (**G**) Anti-SARS-CoV-2 IgM (left) and IgG (right) versus days since symptom start for RD cohort. (**H**) Frequency of PB within nnB cells versus days since symptom start for RD cohort. (**A-E, G-H**) Each dot represents an individual patient or subject, with adult HD in gray circles, adult COVID-19 in shades of mauve indicated by disease severity score, RD in purple circles, Ped COVID-19 in blue circles, with ARDS patients in dark blue, and MIS-C in green triangles. See key, top right. (**A**) Significance determined by unpaired Wilcoxon test between adult HD and each pediatric cohort, and between pediatric groups, with adjustment for multiple comparisons using Benjamini-Hochberg correction; indicated by **** p<0.0001. Lack of notation for specified comparisons indicates no statistical significance. (**B**) Non-parametric trend lines (Theil-Sen) for each pediatric cohort, with Spearman’s Rank Correlation coefficient (ρ) and P value. (**C**) Significance determined by unpaired Wilcoxon test between pediatric cohorts. (**DE**) For MIS-C, paired *t* test P value is shown for the three subjects with repeat draws. (**EH**) Gray shading indicates the derived central 95% adult HD reference interval. (**G**) Non-parametric trend lines (Theil-Sen) with Spearman’s Rank Correlation coefficient (ρ) and P value. Dotted gray line denotes positive assay threshold. See Table S5 for subject numbers per panel.

In the United States, almost all MIS-C patients are seropositive for SARS-CoV-2 specific antibodies, consistent with a delayed clinical presentation (15, 24, 73, 74). Both pediatric COVID-19 and MIS-C had similar frequencies of naive, CD27-IgD-, non-switched memory, and switched memory B cells (Fig. S6A-C). However, given the likely 3-4 week time difference in clinical presentation of MIS-C relative to SARS-CoV-2 infection, we hypothesized that MIS-C patients would be past the peak PB response and would therefore have lower PB frequencies compared to pediatric COVID-19 patients. Instead, PB frequencies were elevated in both pediatric COVID-19 and MIS-C compared to healthy adults, suggesting ongoing B cell responses in both settings (Fig. 4A). As in adults (*12*), the blood PB frequency did not correlate with spike receptor binding domain (S-RBD)-specific IgM or IgG in acute pediatric COVID-19 cohort or in MIS-C (Fig. 4B). PB frequencies were nominally positively correlated with, and naive B cells negatively correlated with, plasma IFNγ (Fig. S6D). Furthermore, a shift from naive toward CD27-IgD- B cells was associated with IL-6. In contrast, there were no significant associations between activated or proliferating T cell subsets and cytokines (Fig. S6E). Together these data suggested that the PB responses were elevated in MIS-C compared to healthy subjects and that B cell responses in MIS-C maintain a similar relationship to the measured plasma cytokines as observed in pediatric COVID-19.

Although total PB frequencies were comparable between pediatric COVID-19 and MIS-C, the differentiation state of the PB was distinct. Specifically, the T-box transcription factors T-bet (*P*=0.051) and Eomesodermin were higher in PB from MIS-C compared to pediatric COVID-19 (Fig. 4C, Fig. S6F). T-bet expression in B cells has been associated with extra-follicular responses, advanced age, autoimmunity, and viral infections (*64*–*67*). These PB T-bet data suggest that, although similar in frequency, the differentiation state of PB responding in MIS-C patients is altered compared to pediatric COVID-19.

We next assessed the T cell component of humoral immunity, Tfh. Like PB, circulating Tfh (cTfh) that express ICOS and/or CD38 increase in blood 1-2 weeks after immunologic challenge (*68*, *69*). Total cTfh frequencies were similar between pediatric COVID-19, MIS-C, and healthy adult donors (Fig. S7A). The frequency of activated cTfh was also similar to healthy adult donors (Fig. S7B). Moreover, total or activated cTfh populations did not correlate with the PB response in either pediatric cohort (Fig. S7C-E). Of note, both the frequency and gMFI of CXCR5 were reduced in pediatric COVID-19 (gMFI only) and MIS-C compared to healthy adults (Fig. S7F). Whether the lower expression of this follicular-homing chemokine receptor could impact coordination of the germinal center and humoral response or be a symptom of follicular disruption is unclear.

The stability of S-RBD-specific antibodies and PB was next examined in the eight patients who underwent repeated blood draws (5 COVID-19, 3 MIS-C). We plotted time from first hospital admission to visualize changes over time and to highlight time from admission for subjects with only 1 research blood draw. In both pediatric COVID-19 and MIS-C, the level of S-RBD-specific IgG and IgM did not change substantially over 15 days, unless patients received convalescent plasma (open circles, Fig. 4D) (*70*). While patients with ARDS remained hospitalized for weeks, the median duration of hospitalization in MIS-C was 8 days. Patients with ARDS had stable antibody responses into weeks three and four of hospitalization, although convalescent plasma treatment may contribute to this antibody, as we previously reported (*70*). In MIS-C, the frequencies of both total PB and Tbet+ PB decreased during hospitalization and after treatment (Table S1, Fig. S1), with PB responses similar to healthy adults by the second draw (Fig. 4E, healthy adult range in gray shading). In pediatric COVID-19, although PB frequencies decreased to the HD range in many cases, in two patients PB frequencies increased during the first 15 days. A similar pattern was observed for Tbet+ PB. Together these data suggest that MIS-C is associated with stable antibody responses over time but with declining PB frequency and a change in PB differentiation state during clinical improvement.

The elevated PB frequencies in MIS-C are perhaps surprising, given that MIS-C is hypothesized to occur weeks after SARS-CoV-2 infection. One possibility is that SARS-CoV-2 infection typically results in a prolonged PB response in the blood. To investigate this possibility, we examined PB frequencies in recovered adult donors (RD) for whom blood samples were obtained 17-69 days after symptoms onset. RD were symptomatic individuals who were diagnosed with SARS-CoV-2 infection by PCR, never hospitalized, and then experienced resolution of all symptoms (Fig. 4F) (*12*). We plotted measures of B cell and humoral immunity relative to symptomatic disease onset, providing a temporal approximation of immunological features in a non-MIS-C cohort over a time frame relevant for MIS-C. As expected, over the course of 17-69 days since symptom initiation, the quantity of S-RBD-specific IgM decreased, whereas S-RBD-specific IgG was detectable and remained relatively stable (Fig. 4G). However, PB frequencies in RD were similar to adult healthy donor PB frequencies (gray bar), even within the first month after symptom start (Fig. 4H), suggesting rapid resolution of the PB response in typical SARS-CoV-2 infection. Although the comparison is limited because age is not matched between MIS-C and RD, the high PB frequencies at clinical presentation for MIS-C suggest a prolonged PB response after initial infection or a delayed initiation of PB responses in MIS-C. In either case, patients with MIS-C resolved these PB features as they clinically improved with treatment including immune modulation (Table S1, Fig. S1).

### Immune perturbations in MIS-C overlapped with severe adult COVID-19 and corrected with clinical improvement

We next tested whether the immunologic differences between MIS-C and pediatric COVID-19 were a reflection of illness severity rather than a reflection of different diseases. To interrogate this question, we re-grouped our pediatric COVID-19 patients into those with minimal versus severe disease, where severe disease was defined as a primary respiratory illness with requirement of positive pressure respiratory support above baseline, including ARDS. We then replotted the data from the first research blood draws (presented in Figs. 1-4) using this severity categorization (Fig. S8A-E). Frequencies of activated (HLA-DR+CD38+ or Ki67+) CD8+ and CD4+ T cells (Fig S8A-B) and activated CX3CR1+ CD8+ T cells (Fig S8D) remained elevated in MIS-C even when compared to the severe subgroup of pediatric COVID-19. There was also a trend toward an increase in PD-1+ and PD-1+CD39+ CD8+ T cells (Fig S8C), but PB were not different (Fig S8E). Although pandemic restrictions prevented contemporaneous acquisition and analysis of samples from healthy pediatric subjects, we also examined selected immunologic features using cryopreserved samples to specifically test whether the healthy adult samples provided a reasonable proxy for some immune features of healthy children (Fig. S8F-J). The adult (ages 22-61) and pediatric (ages 6-12) cryopreserved samples revealed low levels of immune activation that were mostly similar between healthy adults and children. Although future studies comparing pediatric COVID-19 and MIS-C to age-matched healthy children are warranted, these data suggest that healthy adults available here provide a useful control.

**Fig. 5:**
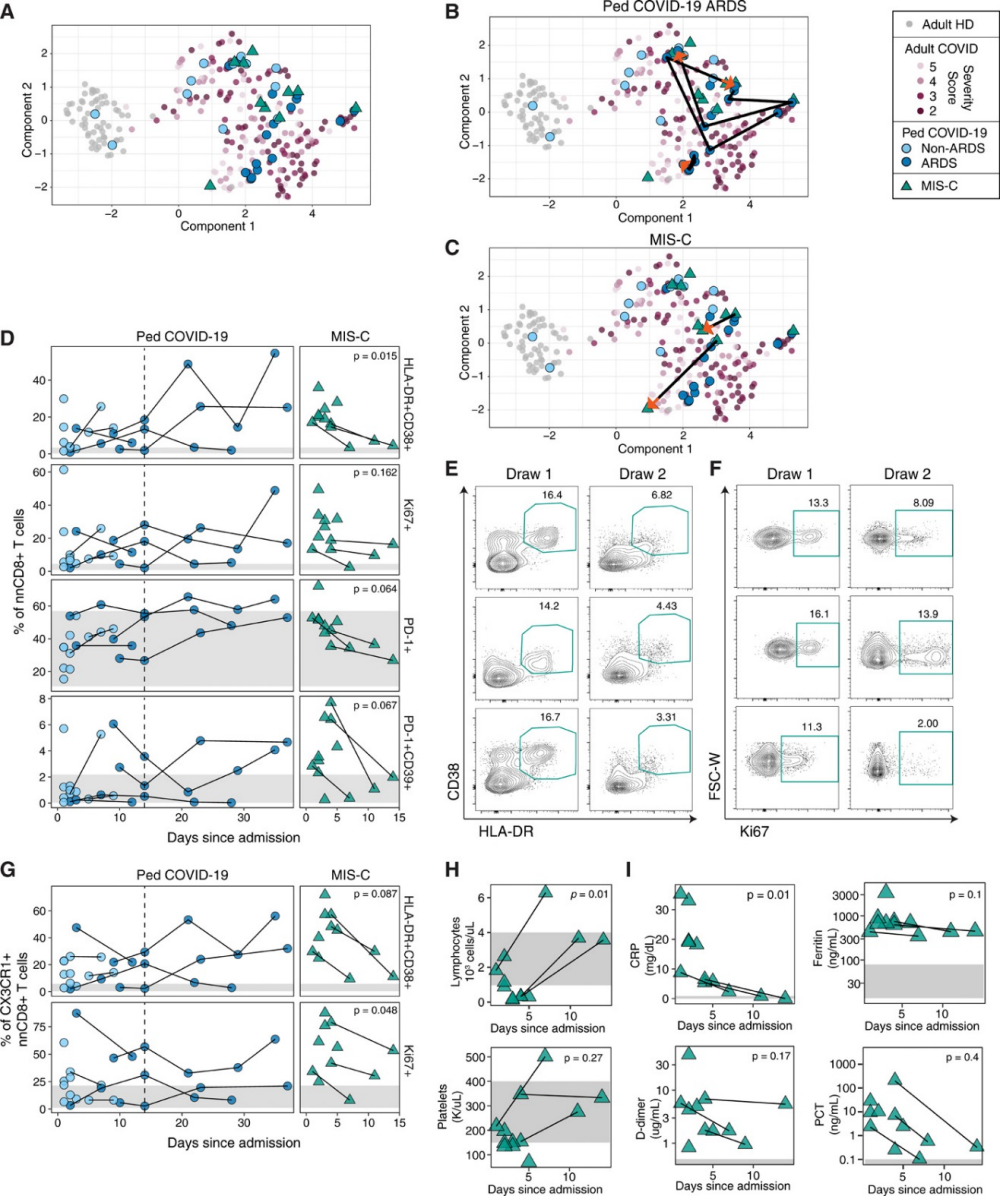
Immune perturbations in MIS-C overlapped with severe adult COVID-19 and corrected with clinical improvement. **(A)** Transformed UMAP projection of aggregated flow cytometry data from PBMC analysis. (**B**) Trajectory in UMAP space for repeat Ped COVID-19 ARDS draws. (**C**) Trajectory in UMAP space for repeat MIS-C draws. (**D**) Activated and proliferating CD8+ T cell populations over days since admission in Ped COVID-19 (left) and MIS-C subjects (right). (**E**) Flow cytometry plots for HLA-DR+CD38+ nnCD8+ for MIS-C patients with repeat draws. (**F**) Flow cytometry plots for Ki67+ nnCD8+ for MIS-C patients, as in (**E**). (**G**) CX3CR1+ HLA-DR+CD38+ and Ki67+ CD8+ T cell populations over days since admission in Ped COVID-19 (left) and MIS-C subjects (right). (**H**) Lymphocyte and platelet counts over days since admission for MIS-C subjects. (**I**) Clinical inflammatory markers over days since admission for MIS-C subjects. (**A-D,G-I**) Each dot represents an individual patient or subject. See key, top right. (**DGHI**) Black lines connect repeat draws for individual subjects. For MIS-C, paired *t* test P value is shown for the three subjects with repeat draws. (**DG**) Gray shading indicates the derived central 95% adult HD reference interval. (**HI**) Gray shading indicates normal clinical laboratory reference ranges for pediatric subjects. See Table S5 for subject numbers per panel.

To ask in a global unbiased manner whether the pediatric immune landscape was distinct from COVID-19 adults, we built a Uniform Manifold Approximation and Projection (UMAP) to distill 207 immune features into two-dimensional space. Immune features were restricted to non-naive T and B cells to eliminate the strongest source of age-associated variation, although features of naive T cells were also examined (Table S4). As reported (*12*), adult HD and COVID-19 adults were immunologically distinct (Fig. 5A). Notably, the immune landscape of pediatric MIS-C and most pediatric COVID-19 patients was generally located with COVID-19 adults rather than adults who were healthy. Indeed, the one pediatric COVID-19 subject without symptoms or clinical evidence of disease clustered with ill adults. However, two pediatric COVID-19 subjects with evident disease clustered more closely with healthy adults. MIS-C patients, in particular, overlapped with adults who were severely ill.

Given differences in clinical trajectory between patients admitted to the pediatric ICU with ARDS versus MIS-C, we next investigated whether the clinical course was mirrored in UMAP high dimensional immunologic space. One patient with ARDS demonstrated a stable immunophenotype over time, with all 5 timepoints in close proximity on the UMAP, whereas two other patients displayed considerable movement in UMAP space indicating temporal changes in immune response (Fig. 5B, Figure S1). We next assessed the trajectory for 2 MIS-C patients in whom repeat samples were available for UMAP (Fig. 5C). Both MIS-C patients moved toward a position enriched for less ill adults (NIH score 4-5) and toward adult HD. The two MIS-C patients were 0 and 3 days from hospital discharge at the second blood draw (Fig. S1). Thus, although the number of patients is small, clinical improvement in MIS-C was associated with global immune landscape changes toward locations associated with less severe disease in adults.

We next asked whether the MIS-C position changes on the UMAP were associated with decreased T cell activation as patients progressed toward hospital discharge. In ARDS, CD4+ T cells initially displayed minimal activation and were similar to healthy adults for HLA-DR, CD38, Ki67 as well as PD-1 and CXCR5 expression (Fig. S9A-C). In contrast, MIS-C patients initially demonstrated more CD4+ T cell activation than healthy adults by most metrics, but this activation resolved over time (Fig. S9A-C). Unlike CD4+ T cells, many features of CD8+ T cell activation and proliferation were elevated compared to the healthy adult range at admission for pediatric COVID-19 and MIS-C patients (Fig. 5D). Furthermore, in patients with prolonged admission due to ARDS, CD8+ T cell activation and proliferation remained elevated over time (Fig. 5D). This pattern contrasted with MIS-C, where HLA-DR+CD38+ CD8+ T cells decreased in the first two weeks of admission (Fig. 5D, Fig. 5F) and PD-1+CD39+ CD8+ T cells fell to the range observed in healthy adults (Fig. 3D, Fig. 5D). The frequency of Ki67+ CD8+ T cells in MIS-C also decreased in the second week of admission, though proliferating CD8+ T cells remained elevated compared to healthy adults for two of three patients (Fig. 5D, Fig. 5F). Similarly, proliferating or activated CX3CR1+ CD8+ T cells decreased as MIS-C patients moved toward hospital discharge (Fig. 5G). In contrast, in ARDS patients where activation or proliferation of CX3CR1+ CD8+ T cells was above the normal range, these CD8+ T cells remained activated over time (Fig. 5G). Thus, the different temporal patterns of CD8+ T cell activation may explain continued co-localization of ARDS with severely ill adults in the UMAP as well as movement of the MIS-C patients toward UMAP locations associated with decreased T cell activation over time.

The immunophenotypic changes in MIS-C corresponded to correcting lymphocyte and platelet counts (Fig. 5H), as well as downtrending CRP, D-dimer, PCT and ferritin (Fig. 5I). However, only CRP declined to the normal range by the second research blood draw. These clinical and cellular measures of immune dysregulation suggested an ongoing resolution of pathology, though resolution remained incomplete near the time of discharge. Together these data revealed a profound immunologic activation in MIS-C that began to resolve as patients were treated clinically (including with immunomodulatory therapy) and recovered from disease.

## DISCUSSION

SARS-CoV-2 associated MIS-C is likely immunologically mediated as MIS-C patients demonstrate markedly elevated clinical measures of inflammation and respond to immune modulation with IVIg and steroids. However, the underlying immuno-pathogenesis of MIS-C remains poorly understood. Here, we performed deep immunologic profiling of 14 children with MIS-C and 16 children with COVID-19 during the phase of illness that required hospitalization. Our studies revealed distinct features of lymphocyte activation and B cell responses that give insights into MIS-C pathogenesis and immunologic shifts over time in pediatric COVID-19.

One of the more striking differences between MIS-C and pediatric COVID-19 was activation of CX3CR1+ CD8+ T cells. CX3CR1 is a chemokine receptor expressed by myeloid cells and lymphocytes that binds the ligand CX3CL1 (*71*). For lymphocytes, CX3CR1 is expressed by cytotoxic, effector-like CD8+ T cells and NK cells and, in particular, expressed by a CD8+ T cell population that can interact with the vasculature with a proposed role in control of persisting and/or reactivating viral infection (*55*, *57*, *72*). Moreover, this CX3CR1-CX3CL1 axis has a role in cardiovascular disease, where CX3CL1 is expressed and presented by vascular endothelial cells to mediate adhesion and then subsequent extravasation of leukocytes (*73*). CX3CL1 expression can be increased by inflammatory cytokines present during viral infections and, in cardiovascular disease, this chemokine increase in the vasculature leads to recruitment of CX3CR1+ T cells (*73*). Thus, the identification of increased activation of CX3CR1+ CD8+ T cells in MIS-C could have relevance to the vascular pathology observed in these patients. Indeed, this immunological phenotype was associated with a requirement for vasoactive support, elevated D-dimer, and decreased platelets in all pediatric patients. These data also point to potential mechanisms of disease in adults. In particular, our studies identify a potential relationship between thrombotic complications in adult COVID-19 patients and activation of CX3CR1+ CD8+ T cells. It will be interesting in the future to assess whether there is a causal relationship between changes in activation of these CX3CR1+ CD8+ T cells vascular disease presentation and/or resolution and whether approaches such as inhibitors of this CX3CR1-CX3CL1 axis might have clinical potential.

The current studies also highlight the potential temporal differences in B cell responses in MIS-C compared to acute pediatric COVID-19 and resolved adult disease. In US cohorts, patients with MIS-C are almost universally seropositive, unlike in pediatric and adult COVID-19 (*23*, *74*–*77*). Seropositivity is consistent with the notion that MIS-C is a delayed event, presenting weeks after initial SARS-CoV-2 infection (i.e., with enough time for antiviral antibody to develop). The substantially elevated PB frequencies we observed in MIS-C are, therefore, perhaps surprising. In adults who recover from COVID-19, PB frequencies return to baseline 2-4 weeks after symptoms resolve, whereas the PB responses in a subset of hospitalized adult COVID-19 patients can be prolonged (*12*). The reasons for increased PB frequency in MIS-C at admission are unclear but could reflect either aberrantly sustained PB production or a newly initiated response. The presence of a higher proportion of T-bet+ PB in MIS-C could be consistent with an aberrant or extrafollicular response (*64*, *66*, *67*), but in other settings T-bet expression in B cells is required to control viral infection (*65*, *78*, *79*). Of note, PB and T-bet+ PB frequencies in MIS-C declined rapidly after hospitalization. This decrease could reflect the idea of a temporally more advanced immune response in MIS-C that resolves with time or reflect a response to immune modulation with IVIG and/or steroids. Together our data support skewed B cell responses in MIS-C, but future studies will be necessary to dissect whether and how germinal center reactions are altered in this novel inflammatory syndrome.

Our findings provide several key insights into the potential drivers of immune pathogenesis in MIS-C. One possibility suggested by our data is continued activation of adaptive immune responses, driven by persisting antigen. In this context, MIS-C may reflect the later stage of a poorly controlled primary infection. Many MIS-C patients have positive PCR tests at presentation (*23*). Eighty-six percent of MIS-C patients were PCR-positive in this study, in part due to an assay that called a positive sample up to a cycle threshold of 45. In this setting of detectable SARS-CoV-2 RNA, CD8+ T cell activation, elevated frequencies of PD-1+CD39+ CD8+ T cells, and robust PB in the blood at clinical presentation in MIS-C are consistent with responses to persisting antigen (*52*–*54*). However, the long pre-symptomatic phase and rapid development of severe MIS-C may be inconsistent with a chronic or indolent infection and suggest a second possibility: an additional trigger occurring ~2-3 weeks after the initial infection with SARS-CoV-2. This new event could either be virologic (e.g., SARS-CoV-2 localizes to a new tissue type) or due to a secondary infectious or auto-reactive trigger. Third, our observation of a sustained PB response with increased expression of T-bet highlights a potential role for antibody in MIS-C pathogenesis. Further studies are required to assess whether such sustained PB responses are SARS-CoV-2 specific or an auto-reactive antibody response. Taken together, studies of antigen specificity will be critical in understanding lymphocyte activation and immune dysregulation in MIS-C.

In summary, deep immune profiling of >200 immune parameters measured in the peripheral blood of pediatric COVID-19 and MIS-C reveal similarities of pediatric COVID-19 patients with adult COVID-19 patients in many respects. However, the activation profiles of MIS-C often aligned more strongly to adults with more moderate-to-severe disease, suggesting a more robust response, with these immune activation patterns resolving over time. Pediatric studies like ours are often limited by a smaller number of subjects available for analysis, and future larger cohorts will be informative. Moreover, the clinical realities of studying such patients result in heterogeneity in treatments received prior to blood draws as well as in timing of the sample availability relative to symptom onset. Understanding immune state before immune modulation will be important in the future to more accurately define immunologic features of MIS-C. Taken together, comparing and contrasting the immune system in distinct clinical presentations of SARS-CoV-2 infection will help guide precision immunotherapeutics in children and may shed light on the pathology of severe disease in diverse COVID-19 patient populations.

## MATERIALS AND METHODS

### Study Design and Human Subjects

The objective of the study was to define blood immune features in pediatric patients with SARS-CoV-2 infection and to evaluate those features in the context of adult COVID-19 samples collected and analyzed in parallel. The pediatric study began in April 2020 and was expanded in May 2020 to include patients with MIS-C.

#### Pediatric Subjects

Patients age ≤18 were approached for consent after testing positive for SARS-CoV-2 by polymerase chain reaction (PCR, cycle threshold <45). MIS-C diagnosis was adjudicated by a multi-disciplinary medical team and was defined as presentation with fever, clinically severe illness, and multisystem organ involvement (>2 of cardiac, renal, respiratory, hematologic, gastrointestinal, dermatologic, or neurologic), along with positive SARS-CoV-2 PCR or serology. Patients with suspected MIS-C and a negative PCR could be enrolled before SARS-CoV-2 serology results were received. The research protocol was approved by the Children’s Hospital of Philadelphia Institutional Review Board. Verbal informed consent was obtained from a legally authorized representative as per the Declaration of Helsinki. Written informed consent was signed by the consenting physician and a copy was provided to participants. Repeat samples were obtained in a subset of patients who remained in the hospital. Some patients were previously described (*17*, *20*, *70*, *75*). Healthy pediatric subjects were enrolled in 2018-2019.

#### Adult subjects

Peripheral blood samples from adults were collected as described (*12*, *14*). COVID-19 subjects were classified according to an ordinal scale: (1) death, (2) hospitalized, on invasive mechanical ventilation or ECMO or both, (3) hospitalized, requiring nasal high flow oxygen therapy, non-invasive mechanical ventilation, or both, (4) hospitalized, requiring supplemental oxygen, (5) hospitalized, not requiring supplemental oxygen or on pre-hospital baseline oxygen, (6) non-hospitalized, but unable to resume normal activities, (7) non-hospitalized, with resumption of normal activities. Healthy donors (HD) were adults with no prior diagnosis or symptoms of COVID-19. Recovered COVID-19 donors (RD) were adults with a prior positive SARS-CoV-2 PCR test who then recovered. The *n* for each cohort varied by assay performed and completeness of clinical or flow cytometric data, see Table S5.

### Clinical data abstraction

For inpatients, clinical data were abstracted from the electronic medical record into REDCap databases. Clinical laboratory data were abstracted as maximum, minimum, or from the date closest to research blood collection (see **T**able S1). Obesity was defined as body mass index > 95th percentile. Adult subjects were categorized by the clinical team as having suspected or confirmed thrombotic complications, defined as deep venous thrombosis, pulmonary embolism, arterial thrombus, myocardial infarction, cerebrovascular accident, and/or continuous hemodialysis circuit clotting. Diagnosis was based on clinical suspicion, and there was no routine screening employed beyond baseline electrocardiogram on ICU admission. All participants or their surrogates provided informed consent in accordance with protocols approved by the regional ethical research boards and the Declaration of Helsinki.

### Blood Processing

Peripheral blood was collected into sodium heparin tubes (BD, Cat#367874) and processed as described (*12*, *14*). Pediatric COVID-19, MIS-C, adult COVID-19, adult recovered, and adult healthy subjects were collected and processed in parallel during the study period.

### Antibody panels and staining

See Table S2 for antibody panel information. PBMC were stained as described (*12*). Whole blood cell staining was performed as described (*14*). See Table S3 for antibody panel information. Pediatric COVID-19, MIS-C, adult COVID-19, adult recovered, and adult healthy subjects were stained in parallel.

### Flow Cytometry

Samples were acquired on a 5 laser FACS Symphony A5 (BD Biosciences). Optimized photomultiplier tube voltages (PMTs) were determined by voltration and tracked over time using SPHERO rainbow beads (Spherotech, Cat#RFP-30-5A). Compensation was performed using UltraComp eBeads for PBMC study (ThermoFisher, Cat#01-2222-42) or BD CompBeads (BD Biosciences, Cat#552843 and 552844). Up to 2x10^6^ live PBMC or 5x10^6^ total events from whole blood were acquired per each sample. Pediatric COVID-19, MIS-C, adult COVID-19, adult recovered, and adult healthy subjects were acquired in parallel.

### Flow Cytometric Analyses

Analysis and visualization was performed in FlowJo (Treestar, version 10.6.2). Major lymphocyte populations were identified as shown in Fig. S2G. Compared to the strategy used in Mathews *et al*. (*12*), an additional gate was added to exclude CD16^bright^ SSC-A^high^ monocyte-like cells. As described (*12*), batch correction was performed for samples acquired before and after a panel change to remove one antibody. A variance stabilizing transform was applied to each of the primary flow features separately (logit for fraction-of-parent features and logarithm for gMFI features), the mean of the second panel was centered to match the first, and the result was inverse-transformed back to the original scale.

### Serology

Enzyme-linked Immunosorbent Assays (ELISAs) were performed using plates coated with the receptor binding domain of SARS-CoV-2 spike, as described (*75*, *80*). Plasma samples were diluted 1:50. If the IgG or IgM concentration was above the lower limit of detection (positive, set at 0.20 arbitrary units), the sample was re-run in at least a 7-point dilution series for quantitation. A dilution series of the monoclonal antibody IgG CR3022 (specific for the SARS-CoV-2 spike protein) was used as a control across plates.

### Plasma Cytokine Analyses

Blood collected into lithium heparin tubes was spun for plasma isolation in pediatric subjects. Cytokine profiling was performed using V-Plex Pro-inflammatory Panel 1 Human Kits (Cat. #K15049D; Meso Scale Diagnostics, Rockville MD, USA). Samples were tested in duplicate and results measured using the QuickPlex SQ120 (Meso Scale Diagnostics). Cytokine measurements were performed from samples drawn at the same time as the corresponding research blood draw or, when obtained separately, values used for correlation were those closest to the research blood draw time point.

### Statistics

Unless otherwise noted, non-parametric tests were used to accommodate for data heterogeneity. Correlation was quantified by Spearman’s rank correlation coefficient (ρ). Associations between ordered features were evaluated by Spearman’s rank correlation test. Associations between discrete unordered features were evaluated by Fisher’s exact test. Associations between mixed ordered versus unordered features were performed by unpaired Wilcoxon test. Discrete unordered features with > 2 categories were expanded into binary “dummy” variables prior to testing by the methods described above. To test for a difference in the Spearman rank correlations between features in Fig. 2G, a non-parametric permutation test was used. Briefly, all underlying paired data were held fixed and a difference statistic, Δρ = ρ_MISC_ - ρ_COVID_, was computed under the exact null distribution derived by all unique permutations of the binary COVID-19 versus MIS-C label. Trajectory analysis was performed by paired *t* test across all MIS-C patients with paired sequential blood draws. All other paired analyses were performed by non-parametric paired Wilcoxon test. All tests were performed two-sided with a nominal significance threshold of P < 0.05. In all cases of multiple comparisons, false discovery rate (FDR) correction was performed using the Benjamini-Hochberg procedure. In heatmaps, black boxes indicate significance at the FDR < 0.05 threshold. Lack of black box indicates failure to meet this cut-off.

## Supplementary Materials

immunology.sciencemag.org/cgi/content/full/6/57/eabf7570/DC1 Fig. S1. Treatment and sampling course for pediatric cohort over hospital stay. Fig. S2. Innate immune compartment in MIS-C is broadly comparable to Pediatric COVID-19 Fig. S3. T cell proliferation in MIS-C is greater than in COVID-19 Fig. S4. NK and CD8+ MAIT cell activation in MIS-C is greater than in COVID-19 Fig. S5. Vascular patrolling CX3CR1+ populations in MIS-C are uniquely proliferative in MIS-C Fig. S6. A transition towards B cell memory subsets from naive is associated with IFNγ in both MIS-C and pediatric COVID-19 Fig. S7. Total and activated cTfh frequencies are not associated with PB responses in either pediatric cohort. Fig. S8. T cell activation in MIS-C is elevated compared to severe pediatric COVID-19 disease. Fig. S9. Features of CD4+ T cell activation start to normalize over time, coincident with treatment and recovery from disease. Table S1: Patient Characteristics Table S2: Panel for Peripheral Blood Mononuclear Cell Flow Cytometric Staining Table S3: Lineage Panel for Whole Blood Flow Cytometric Staining Table S4: Flow cytometry features included in UMAP analysis Table S5: Sample n per figure panel Table S6: Raw Data File (excel spreadsheet)
